# *In vitro* characterization of amino acid digestibility and fermentative properties of a specific hydrolyzed yeast, and its *in vivo* effects on growth performance and fecal microbiota in weanling piglets

**DOI:** 10.3389/fnut.2025.1596561

**Published:** 2025-07-14

**Authors:** Fernando Bravo de Laguna, Caroline S. Achard, Lysiane Dunière, Elsa Parmentier, Katia Helmja, Bruno Bertaud, Pierre Lebreton, David Saornil, Eric Chevaux, Mathieu Castex, Emmanuelle Apper

**Affiliations:** ^1^Lallemand SAS, Blagnac Cedex, France; ^2^UMR 454 MEDiS, Université Clermont-Auvergne, INRAE, Saint Genès Champanelle, France; ^3^TFTAK, Tallinn, Estonia

**Keywords:** hydrolyzed yeast, *Saccharomyces cerevisiae*, protein digestibility, amino acids digestibility, absorption kinetics, weanling piglets, microbiota, performance

## Abstract

Weaning is a stressful event that is often accompanied by anorexia, risk of diarrhea, and development of intestinal disorders, making it crucial to provide highly digestible and palatable diets. Novel functional protein sources are being developed to be included in diets fed to weanling pigs. We evaluated *in vitro* and *in vivo* the properties of a recently developed hydrolyzed yeast protein source (Yela ProSecure; YPS; Lallemand SAS, Blagnac, France). The objectives were (1) to evaluate *in vitro* amino acids (AA) digestibility; (2) to assess, *in vitro,* the impact of the product’s insoluble fraction (YPSi) on the fermentative activity of piglet fecal microbiota; and (3) to test the effects of two inclusion levels (2.5 and 6%) on growth performance and fecal microbiota in weanling piglets. The total AA availability after 3 h of digestion was 76.6%, reaching 89.8% after 48 h. YPSi induced high gas and short-chain fatty acids production. In the *in vivo* experiment, a significant difference in body weight was observed on day 18 (*p* < 0.001) post-weaning and on day 40 (*p* < 0.05), with piglets in both YPS treatments being heavier than control piglets. A higher average daily feed intake was observed between weaning and day 18 (*p* < 0.01) and overall (*p* < 0.05) in both YPS treatments, and an improved feed conversion ratio was observed in both YPS treatments between weaning and day 18 (*p* < 0.001). Moreover, YPS significantly modulated the fecal microbiota composition after 2 days and 16 days of treatment, whereas no lasting effect was evidenced on day 40, namely after 19 days of withdrawal from the diet. Lower relative abundance (RA) of *Campylobacterota* (*p* adj. < 0.05) was evidenced in YPS groups compared to the control group. Furthermore, several members of the *Lactobacillaceae* family, annotated as *L. amylovorus*, *L. mucosae,* or *L. reuteri,* as well as *Faecalibacterium prausnitzii*, showed higher RA in YPS groups. To conclude, adding YPS to the diet of weanling piglets increased growth performance, probably due to nutrient absorption in the small intestine and its functional role on gut microbiota. Those results suggest complex interconnections between host and microbiota and emphasize the need to consider the holobiont theory when formulating a diet.

## 1 Introduction

In swine production, weaning is a stressful event often associated with anorexia, increased risk of diarrhea, and the development of intestinal disorders. This makes it crucial to provide highly digestible and palatable diets. Proteins are a key component of these diets, as they supply amino acids (AA) necessary for optimal growth and health. However, the increasing global human population is driving increased demand for animal production, which in turn is putting pressure on the protein market and raising concerns about a “protein gap.” Additionally, environmental and sustainability issues related to feeding animal-derived proteins to food-producing livestock have led to an increase in the use of land for cultivating plant-based protein sources. Competition with human-edible proteins, pressure on agricultural land, sustainability challenges, and climate changes are limiting factors for the development of conventional protein sources (1). In this context, novel qualitative and sustainable protein sources that can be produced in large quantities are being studied to formulate novel piglets’ diets.

Single-cell proteins produced from yeast constitute one category within the proposed protein alternatives. Yeast and yeast derivatives contain, among others, significant quantities of AA, mannans, *β*-glucans, chitin, and nucleotides (2, 3) and are notably used as feed additives to improve growth performance and immune response in weanling pigs (4–6). Yet, yeast can be subjected to a technological process with the objective of making its nutrients more available for the animals. Hydrolyzed yeasts are obtained through specific digestion of inactivated yeasts by both endogenous and exogenous enzymes (7). During the process, the cell wall is broken, and peptides and nucleic acids are fragmented, resulting in highly digestible nutrients. In addition to the direct nutritional value of these components, a beneficial effect on animal health and performance can be expected due to the peptide and fiber composition as well as the numerous metabolites comprised in yeast (8, 9). However, depending on the process applied, the enzymes used to hydrolyze the yeast, and the yeast strain, each hydrolyzed yeast may have its own characteristics (10), with likely different effects.

A specific process has been developed to produce a novel hydrolyzed yeast protein source (Yela ProSecure; YPS; Lallemand SAS, Blagnac, France) to be included in diets fed to weanling pigs. Limited data about the nutritional and functional value of this yeast protein source have been published yet. A series of experiments were conducted with the following objectives: (1) to appraise the *in vitro* AA digestibility; (2) to assess *in vitro* the impact of the insoluble fraction (YPSi) on the fermentative activity of piglet’s fecal microbiota, and (3) to test the effect of two inclusion levels (2.5 and 6%) on weanling piglets’ performance and fecal microbiota. We hypothesize that a protein source containing small peptides and free AA and exhibiting rapid digestion kinetics may enhance AA absorption synchronicity in the upper gut, stimulating feed intake and ultimately improving growth performance. Additionally, the indigestible fraction may influence gut health and gut microbiota composition, conferring an additional lever to optimize piglets’ performance and health.

## 2 Materials and methods

### 2.1 Yela ProSecure characteristics

**Figure 1:**
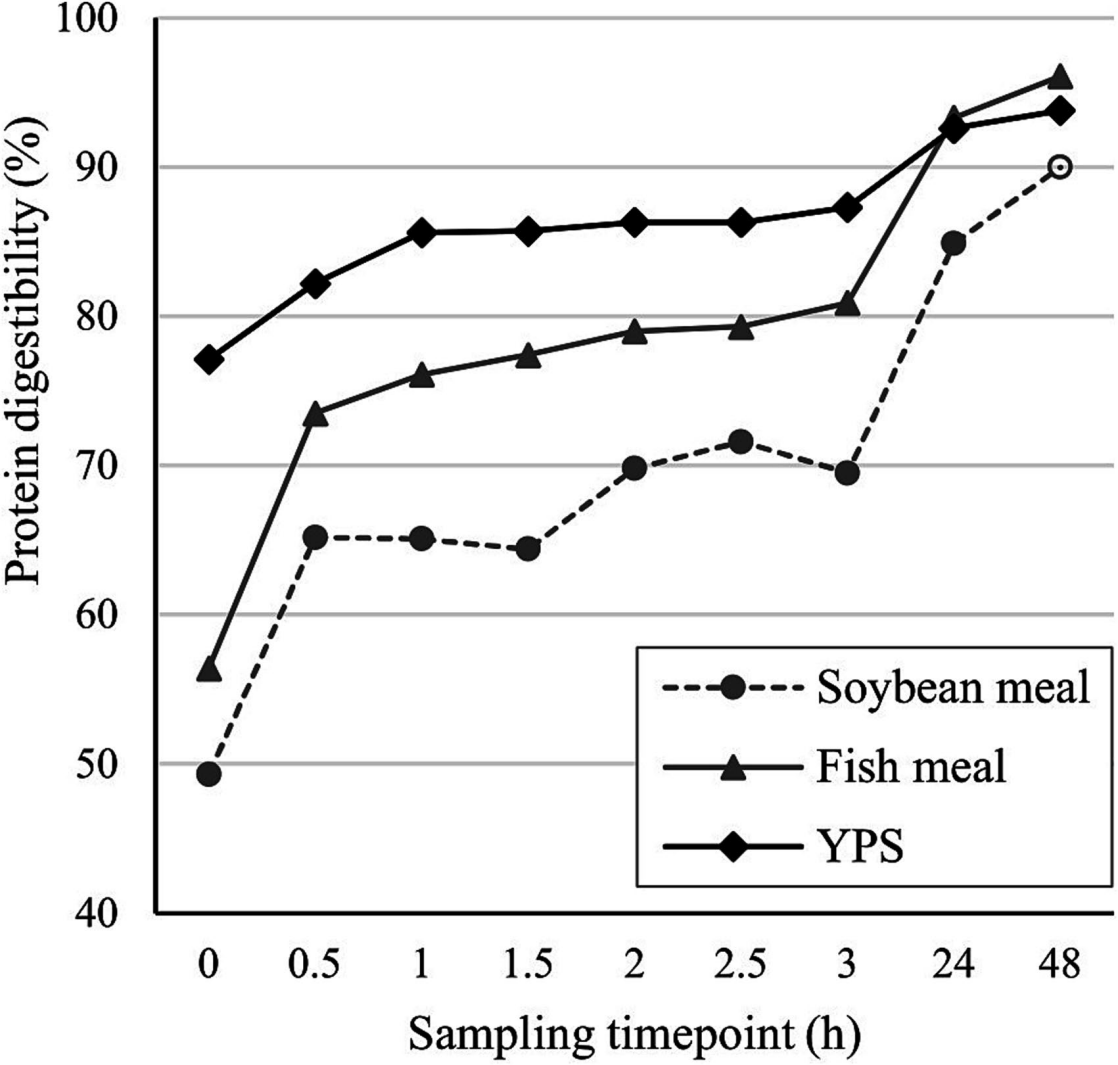
*In vitro* protein digestibility (%) of the tested raw materials.

Yela ProSecure (YPS; Lallemand SAS, Blagnac, France) is a specifically designed hydrolyzed yeast obtained from the fermentation of non-GMO *Saccharomyces cerevisiae* yeast derived from sugar cane ethanol production. It is a feed material produced through a controlled hydrolysis process at high temperatures and controlled pH, which optimizes the cracking of the yeast cells through the effect of endogenous and specifically selected exogenous enzymes. The composition of a representative batch of YPS was compared with two common protein sources used in swine: soybean meal (SBM) and fish meal (FM), both raw materials provided from a Spanish commercial feed meal (Supplementary Table 1). The characterization of YPS as a protein source was performed at the Institut Mutualisé pour les Protéines Vegétales (IMPROVE, Dury, France), based on (11). The protein digestibility of YPS is presented in Figure 1 and compared with SBM and FM.

### 2.2 *In vitro* total AA digestion kinetics

The analysis of AA *in vitro* digestion kinetics of YPS was performed at the TFTAK research institute (Tallinn, Estonia). The *in vitro* procedure was based on the method described by (11), modified by (12), and focused on the digestion kinetics over 48 h. For the stimulation of gastric digestion, 1 g of the sample was incubated in a 100-ml conical flask with a phosphate buffer (25 ml, 0.1 M, pH 6.0) and an HCl solution (10 ml, 0.2 M). The pH was adjusted to 4.5 with 1 M HCl or 1 M NaOH, instead of 2.0, to be more reflective of the condition of the stomach of piglets at weaning (13). Freshly prepared pepsin solution (1 ml, 10 mg/ml, at 2000 FIP-U/g, Merck KGaA, Darmstadt, Germany) and chloramphenicol at 0.5% (A1806, AppliChem GmbH, Darmstadt, Germany) were added and each conical flask was covered with a rubber stopper and placed at 39°C for 6 h in an incubator (KS 3000 i control, IKA, Staufen, Germany) under constant magnetic stirring (190 rpm). Following the incubation with pepsin, the protein digestion in the intestine was simulated by adding 10 ml of phosphate buffer (0.2 M, pH 6.8) and 5 ml of 0.6 M NaOH to the samples, and the pH was adjusted to 6.8 with 1 M HCl or 1 M NaOH. Freshly prepared pancreatin solution (1 ml, 50 g/L, P-1625, Sigma Aldrich, Burlington, MA, USA) was added and the incubation was continued at 39°C under constant magnetic stirring (190 rpm). The incubation times with pancreatin were 0, 3, and 48 h. To correct for the AA of the enzymes added, blanks (triplicates) without protein sources were analyzed. The samples taken during the sequential incubation with pepsin and pancreatin and from blanks were cooled on ice for 10 min and then centrifuged (10 min, 14,000 rpm, Mikro 120, Andreas Hettich GmbH, Tuttlingen, Germany) to separate the soluble protein fraction from the insoluble protein fraction.

The total AA content of the supernatant containing the soluble protein fraction was determined after acid hydrolysis for 22 h at 105°C with HCl 6 N and 1% phenol solution under nitrogen. Twenty microliters of the sample were mixed with 20 μL of in-house produced isotopically labeled biomass as an internal standard before acid hydrolyses. After the hydrolysis process, 200 μL of borate buffer (1 g Na-tetraborate and 1.224 g of boric acid in 100 ml MilliQ water) were added to each sample vial. Finally, samples were filtered through a 0.2-μm Polytetrafluoroethylene filter (Millipore) before the derivatization procedure. The liberated AA was derivatized by AQC (6-Aminoquinolyl-N-hydroxysuccinimidyl carbamate, Synchem UG & Co. KG. Felsberg, Germany). Seventy microliters of borate buffer were mixed with 10 μL of the sample. Then, a derivatization agent with a concentration of approximately 3 mg/ml was added, and the reaction mixture was incubated at 55°C for 10 min. Total AA was then analyzed by liquid chromatography-mass spectrometry (LC–MS/MS; ACQUITY UPLC system equipped with column Acc-Q-Tag Ultra RP 1.7 μm, 2.1 mm × 100 mm and coupled with Waters Quattro Premier XE, Waters Corp., Milford, MA, USA). The flow rate was 0.3 ml/min, with mobile phase A waters AccQTag Eluent A and mobile phase B acetonitrile containing 1% formic acid. In brief, the applied gradient mode was as follows: 0.1% of eluent B, running for 0–1.26 min; 0.1–9.1% B for 1.26–13.39 min; 9.1–21.2% B for 13.39–18.06 min; 21.2–59.6% B for 18.06–18.76 min; 59.6–0.1% B for 18.76–20.37 min, and then 0.1% of B for 20.37–23.33 min. The % AA availability was calculated by the ratio of the sum of total AA in the soluble fraction after incubation and the sum of total AA in the original sample (AA Availability (%) = Total AA in soluble fraction after incubation/Total AA in original sample).

### 2.3 *In vitro* fermentative activity in intestinal condition

#### 2.3.1 Production of insoluble fraction of YPS

An insoluble fraction of YPS (YPSi) was produced. In brief, after rehydration of the YPS product overnight at room temperature (at 15% dry weight in osmosed water), the insoluble fraction was obtained and washed again with osmosed water by 2-step centrifugation (Thermo Multifuge X3FR, 4,200 rpm, 15 min). The crude protein content of the YPSi extract used in this experiment was determined based on the Kjeldahl method and fiber composition according to the Prosky method (14, 15). The pellet was then dried at 80°C for 2 h to obtain a dry matter of 96% and was manually ground. The product obtained was then tested *in vitro* as follows.

#### 2.3.2 Preparation of incubation bottles

Incubations were performed under strictly anaerobic conditions at 39°C to mimic digestive tract conditions. Fecal samples from four visually healthy male piglets were taken a few days after weaning on a commercial farm in Spain. Only males were selected because, due to the proximity between the anus and the vagina, samples from females are more likely to be contaminated with urine. The diet fed to the sampled piglets was based on wheat, barley, extruded barley, extruded rice, and soy protein concentrate; additionally, they had not been fed probiotics or administered any medication prior to sampling. Fecal samples were transported in dry ice to the lab and stored at −80°C until analysis when they were thawed and gently mixed before pooling. Pooled feces were added at a final concentration of 2.5% (w/v) into an anaerobic dilution medium (16) in 125 ml Balch bottles according to the experimental treatments. All media were prepared and used according to Hungate (16) and modified by Niderkorn et al. (17). Each experimental step was conducted under anaerobiosis, with constant 100% O_2_-free CO_2_ gas flux. Media containing resazurin, as a colorimetric indicator of O_2_ contamination, were dispensed in flasks specifically designed for anaerobiosis microorganisms’ cultivation. After inoculum addition in each flask, a 3- to 5-min of 100% O_2_-free CO_2_ gas flux was allowed to remove the maximum of O_2_ that could have been accidentally introduced. As correct amounts of fermentative activity were noted, this model was considered as strictly anaerobic as possible. Three independent incubation series were carried out with three treatments: Control, addition of 10 g/L of YPSi, and addition of 10 g/L of inulin (positive control, Sigma Aldrich, Saint-Louis, MI, USA). Three non-inoculated, non-incubated replicates were used for timepoint 0 h determination. The concentration of inulin was set up at 10 g/L according to Uerlings et al. (18). Both inulin and YPSi products were added in excess to provide non-limiting nutrients for the fecal microbiota as they represented the only source of carbon and energy supplemented to the incubation bottles, and no feed was added. Incubation was performed under anaerobiosis at 39°C with 150-rpm shaking and with technical triplicates per timepoint (8 h and 24 h).

#### 2.3.3 Incubation conditions and measured parameters

For each bottle, gas production during fermentation was measured with a glass syringe, and the gas composition was analyzed through microGC (MicroGC Fusion, Chemlys, Vénissieux, France). Gas molar concentration was calibrated using a certified gas standard mixture (Messer, France) containing CH_4_, O_2_, H_2_, CO_2_, and N_2_. A sample of 2 ml of each incubated bottle was collected for short-chain fatty acids (SCFAs) and branched-chain fatty acids (BCFAs) analysis, centrifuged for 10 min at 10000 rpm; 800 μL of digestive fluid supernatant were then added to 500 μL of 0.5 N HCl containing 2% (w/v) metaphosphoric acid and 0.4% (w/v) crotonic acid before storage at −20°C. The samples were further analyzed using gas chromatography (19) with a wall-coated open-tubular fused silica column (0.25 mm ID 25 m) coated with CP-Wax 58 (FFAP)-CB. Nitrogen was used as carrier gas with a split system. The temperature of the column was set at 130°C for 1 min, increased at a rate of 15°C/min to 220°C, and then held for 2 min. The injector and flame ionization detector were set at 220 and 250°C, respectively. Patulin was analyzed by HPLC using a Nucleosil C18 column and UV detection (275 nm). Sulfhydryl groups in the fermentation were detected with 5,5-dithiobis (2-nitrobenzoic acid; DTNB), following the method of Ellman (20). N-NH_3_ and lactate determination were performed using the Ammonia Assay Kit (Rapid) and D-lactate Assay kit (Megazyme, Wicklow, Ireland) according to the manufacturer’s recommendations.

Duplicate samples of 1 ml for each treatment were collected after incubation and 10-fold diluted (v/v) in anaerobic mineral solution for cultivable microbial analysis. Total strict anaerobes were enumerated in a complex medium containing clarified rumen fluid (21), prepared, dispensed, and inoculated using strictly anaerobic techniques (16) with O_2_-free CO_2_ gas. Bacterial growth was observed visually in culture tubes of each dilution inoculated in triplicate after 72 h of incubation at 39°C, and numeration was determined using the most probable number (MPN) estimation with the McGrady tables (22). Total facultative anaerobes were cultivated by inclusion into G20 agar medium containing peptone (15 g/L), tryptone (10 g/L), yeast extract (5 g/L), glucose (10 g/L), and agar (15 g/L) with pH adjusted to 7.7. Colonies were counted after 2 days of incubation at 39°C. Enterobacteria and putative lactic acid bacteria were, respectively, cultivated on violet red bile glucose agar medium (Biokar diagnostics, Allonne, France) and on Man–Rogosa–Sharpe agar medium (Roth, Karlsruhe, Germany) adjusted to pH 5.5. Agar plates were, respectively, incubated for 1 and 2 days at 39°C, and the developed colonies were then counted. Bacterial count, for the same study, was expressed as log_10_ CFU/g of feces.

### 2.4 *In vivo* trial

#### 2.4.1 Animals and experimental design

A post-weaning performance trial was conducted at the Centro de Pruebas de Porcino del Instituto Tecnológico Agrario de Castilla y León (CPP-ITACyL; Hontalbilla, Segovia, Spain). The experiment was approved by the Ethical Committee in Animal Research of ITACyL, with number 2019/41/CEEA-ITACYL. In the experiment, 96 Iberian × Duroc weanling piglets weaned at 28 days of life with an average body weight (BW) of 7.30 ± 0.738 kg (mean ± SD) started the trial. They were allocated by sex in 24 pens in total, distributed in four rooms, in groups of four piglets per pen. Pens were allotted to three treatments (Supplementary Table 2), yielding eight replicate pens per treatment, and the average initial BW was balanced per treatment and pen. The piglets followed a three-phase feeding program immediately post-weaning: phase 1, between days 0 and 2 to ensure a smooth transition to the nursery period; phase 2, between days 3 and 18; and phase 3, between days 19 and 40. Three dietary treatments were considered: control (CON), YPS 2.5 (CON + YPS at 2.5% during phase 2), and YPS 6 (CON + YPS at 6% during phase 2). In phases 1 and 3, all piglets were fed the same diet without YPS.

The piglets had free access to feed and water throughout the experimental period. The experimental diets of phase 2 (Table 1) were isocaloric and isoenergetic and were formulated to meet or exceed requirements (23). YPS was included at the expense of SBM based on its nutrient composition (Table 2).

**Table 1:** Experimental diets (%, as fed) used in the zootechnical trial.

Ingredients	CON	YPS 2.5	YPS 6
Wheat	30	30	30
Corn	29.26	27.77	26.11
Soybean meal 47%	19.17	16.89	14.3
Barley	8.0	9.37	10.34
Concentrate	2.5	2.5	2.5
Soy oil	1.81	1.8	1.8
Straw (NaOH)	1.5	1.5	1.5
Dicalcium phosphate	1.02	0.86	0.64
Extruded Rice/Oat/Corn (40/35/25)	1	1	1
Whey/Fat 50%	1	1	1
Sweet whey	1	1	1
L-Lys 50% liquid	0.77	0.74	0.67
Calcium carbonate	0.58	0.69	0.83
Mineral Premix	0.5	0.5	0.5
Salt	0.4	0.4	0.4
Tryptophan 25%	0.35	0.34	0.33
Met liquid	0.28	0.28	0.27
Aroma	0.2	0.2	0.2
L-Threonine	0.18	0.16	0.13
Middle chain monoglycerides	0.15	0.15	0.15
Organic acids mix	0.10	0.10	0.10
Coated Na butyrate	0.10	0.10	0.10
Valine	0.09	0.09	0.08
Mold inhibitor	0.05	0.05	0.05
Yela ProSecure	-	2.5	6.0
Nutrients			
Moisture	10.7	10.5	10.2
Crude protein	17.0	17.0	17.2
Lysine	1.25	1.25	1.25
Crude fiber	3.36	3.44	3.53
Ether extract	4.24	4.37	4.57
Starch	42.71	42.44	41.84
Ash	4.89	4.88	4.87

YPS, Yela ProSecure.

**Table 2:** Proximate analysis, amino acids, and B-vitamins composition of Yela ProSecure used in the performance trial (as fed).

Nutrient	Content
Crude protein	42.7%
Crude fat	2.77%
Ash	5.79%
Crude fiber	4.95%
Total dietary fiber	38.90%
Insoluble dietary fiber	33.45%
Soluble dietary fiber	5.45%
Ala	2.84%
Arg	1.65%
Asp/Asn	4.27%
Cys	0.40%
Glu/Gln	4.64%
Gly	1.73%
His	0.77%
Ile	1.71%
Leu	2.82%
Lys	3.09%
Met	0.70%
Phe	1.59%
Pro	1.46%
Ser	2.34%
Thr	2.14%
Trp	0.55%
Tyr	1.37%
Val	2.14%
Vit B1	11.6 mg/kg
Vit B2	24.7 mg/kg
Vit B3	70.4 mg/kg
Vit B4	5,260 mg/kg
Vit B5	81.4 mg/kg
Vit B6	16.2 mg/kg
Vit B7	1,151 mg/kg
Vit B8	511 μg/kg
Vit B9	420 μg/kg
Vit B10	4.40 mg/kg
Vit B12	7.04 μg/kg

#### 2.4.2 Measurements and fecal sampling

Piglets were individually weighted at weaning, on day 18, and at the end of the trial; average daily gain (ADG) was calculated per pen. Feed intake was recorded per pen between weaning and day 18 and from day 19 until the end of the trial, based on the remaining feed at the end of each phase. Average daily feed intake (ADFI) was calculated per pen. The feed conversion ratio (FCR) was calculated from ADFI and ADG. Fecal samples were taken from 16 piglets per treatment group, two piglets per pen, of similar BW on the first sampling day. The same piglets were sampled on days 4, 18, and at the end of the experimental period. Technicians reported if piglets suffered from diarrhea or were healthy. The piglets were held by a technician, and the rectum was stimulated with a sterile swab until defecation. Feces were then placed in a 5-ml cryovial and stored at −80°C. At the end of the experiment, all the samples were transported in dry ice to the lab and stored at 0°C upon analysis.

#### 2.4.3 Microbiota analyses using 16S rRNA gene sequencing

Microbial DNAs were extracted from 20 to 30 mg of fecal samples using Quick-DNA Fecal/Soil Microbe Kit™ (ref D6010, Zymo Research, Freiburg, Germany) according to the manufacturer’s instruction. A 15-min bead beating step at 30 Hz was applied using a Retsch MM400 Mixer Mill. The hypervariable V3-V4 regions of the 16S rRNA gene were targeted for sequencing using the primer set 341F 5′-CCTACGGGAGGCAGCAG-3′ and 806R 5′-GGACTACNVGGGTWTCTAAT-3′. The SuperHotTaq DNA polymerase (Bioron, Römerberg, Germany) was used for the library preparation. High-throughput sequencing was performed on a MiSeq sequencer at the GeT-PlaGe core facility (METYS, INRAe Transfert, Toulouse, France). MiSeq Reagent Kit v3 was used according to the manufacturer’s instructions (Illumina Inc., San Diego, CA, USA). Bioinformatics analyses were performed using the GenoToul bioinformatics facility (Toulouse, France). Sequences were processed using DADA2 pipeline (24) with R software (version 4.1): The FilterAndTrim function was applied with the options trimLeft = c (17,20), truncLen = c(250,235), maxN = 0, maxEE = c (2,3), and truncQ = 2. The Amplicon Sequence Variants (ASVs) were defined using pool = “pseudo” option. Taxonomic annotation was performed using the assignTaxonomy and assignSpecies functions with the SILVA SSU database (version 138). Annotations at the species level were only kept for ASVs exhibiting a 100% identity match. For higher taxonomic ranks, bootstrap values of 50, 70, 80, and 90 were applied for phylum, class, order, family, and genus levels, respectively. Prior to diversity analysis, differential analyses, and functions prediction, a rarefaction step was applied to avoid bias due to differences in sequencing depth (9,507 sequences/sample). A phylogenetic tree was constructed, and weighted and unweighted UniFrac distances were calculated using the FROGS pipeline (version 4.0) supported on the Genotoul-Sigenae Galaxy server (25). Alpha-diversity was estimated using a number of observed species (richness), as well as Pielou, Faith’s PD, and Shannon indices. Beta-diversity was calculated using Bray–Curtis dissimilarity, as well as weighted and unweighted UniFrac distances, with a vegan R package. Phylum, family, and genus relative abundance tables were generated from the ASV rarefied count table, normalized by total sum scaling.

#### 2.4.4 Functional inference

The functional inference was performed using PICRUSt2 software (26–28) with the FROGS pipeline (25). The Nearest Sequenced Taxon Index threshold was set to 0.8 for prediction. Function abundance tables were generated using the Kyoto Encyclopedia of Genes and Genomes Orthology (KO) database. Kyoto Encyclopedia of Genes and Genomes (KEGG) pathways abundance tables were inferred using MinPath (29).

### 2.5 Statistical analysis

SPSS Statistics 26.0 software (IBM, Armonk, NY, USA) was used to analyze performance data. R (version 4.1) and RStudio software were used for all other analyses. Finally, SAS (SAS Institute Inc., Cary, NC) was used to analyze the *in vitro* fermentative activity data. For all the parameters, a *p*-value lower than 0.05 was considered significant, and a *p*-value between 0.05 and 0.1 was considered a trend. An adjusted *p*-value lower than 0.1 was considered significant.

The effects of Treatment (Control, YPSi, or inulin) and Time factors and their interaction were analyzed for *in vitro* fermentative activity data using a mixed model with repeated time and multiple comparisons with Tukey’s adjustment. Normality and homoscedasticity were assessed for all parameters prior to analysis.

Performance data were analyzed according to a general linear model with room, sex, treatment, and their interactions as main effects. Initial BW was used as a covariate. The variability of data was expressed as the standard error of means (SEM). Fecal samples collected for microbiota analyses were scored as “healthy” or “diarrheic” according to a visual examination of the sample. A binomial logistic regression was performed to assess the effect of sampling day and treatment on suffering from diarrhea.

Dissimilarities in microbial composition were tested using multivariate ADONIS2 function (vegan package), accounting for the effects of sampling day, treatment, and their interaction. For taxonomic data analyses, a centered log-ratio transformation was applied to the relative abundance data as advised for compositional data (30). Zero values were replaced by a pseudo count (dataset minimum value divided by 10).

For univariate analyses, a mixed linear model was applied, accounting for the sampling day, treatment, and their interaction as fixed effects and the animal as a random effect. The normality of the model residues was assessed using a Shapiro test. Normality was considered acceptable when *p* > 0.01, and ANOVA with Satterthwaite correction of the degree of freedom was performed using the lmerTest package, followed by emmeans *post-hoc* tests. Alternatively, non-parametric tests were applied to test the effects of sampling day and treatment (Friedman test) and the combination of sampling day and treatment (Kruskal–Wallis test). *Post-hoc* tests were carried out with a Conover test. *p*-values were adjusted using the Benjamin-Hochberg procedure. Differential analyses were applied to features detected in at least half of the samples of at least one group.

Multivariate analyses and feature selection were applied to ASV data for each sampling day separately (MixOmics package, Rohart et al., 2017). Sparse partial least square discriminant analysis (sPLS-DA) allows identifying ASVs that contribute the most to discriminate the samples according to the treatment, whereas the sparse partial least square (sPLS) regression allows selecting ASVs that covary with performance parameters of interest. To assess whether the treatment groups could be discriminated against, partial least square discriminant analysis (PLS-DA) was first performed. Features that represented more than 0.01% of the total reads and detected in at least six samples in at least one treatment group were kept for this analysis. Iterative cross-validation (perf function) was used to assess the robustness of the discrimination. The calculated error rate was used to validate the discrimination, with a mean error rate lower than 50% considered acceptable. Sparse PLS-DA was then applied to select the most discriminant ASVs. The number of components, i.e., new variables created as linear combinations of ASVs, and the number of ASVs to keep in the final model were optimized based on the calculated error rate. Features that represented more than 0.01% of the total reads and detected in at least 10 samples for a given timepoint (i.e., 639 and 574 ASVs on days 4 and 18, respectively) were kept for PLS regression between the microbiota dataset and ADG values. R2 and Q2 values were used to evaluate the PLS model, namely the “goodness of fit” and the “goodness of prediction,” respectively (perf function, Mfold option with folds = 12 and nrepeat = 50), and choose the optimal number of components. Sparse PLS was applied to select the most relevant ASVs. The number of ASVs to keep in the final model was optimized based on the mean absolute error (MAE) criterion.

## 3 Results

### 3.1 *In vitro* characterization of total AA digestion kinetics from YPS

**Figure 2:**
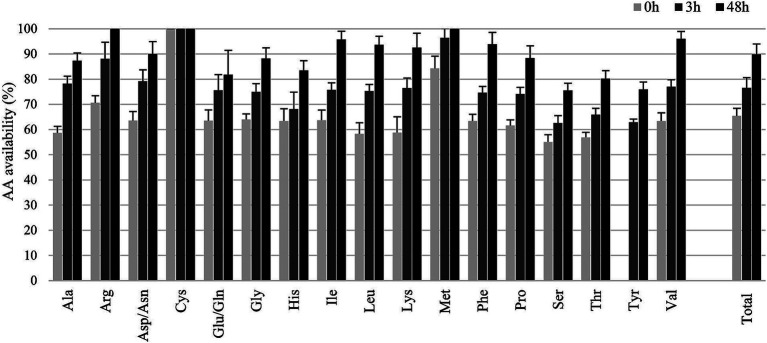
Total amino acids availability (%; mean ± SD) of Yela ProSecure as obtained after *in vitro* digestion.

To estimate the total AA intestinal availability from YPS, three production batches of YPS were analyzed. The average of three technical replicates per sample and timepoint is presented in Figure 2 (data in Supplementary Table 3). Total available AA increased between 0 and 48 h of digestion, reaching 76.6% ± 4.03% after 3 h and 89.8% ± 4.18% after 48 h. After 3 h, AA availability ranges between 62.6% ± 2.93 and 99.9% ± 0.21% observed for Tyr and Cys, respectively.

### 3.2 Effect of YPS insoluble fraction on *in vitro* piglets’ fecal microbial activity

Crude protein and fiber composition of insoluble YPSi are presented in Table 3.

**Table 3:** Protein and fiber composition of insoluble Yela ProSecure used to evaluate the *in vitro* effect on fecal microbial fermentative activity and comparison with Yela ProSecure.

Item, %	YPSi	YPS
Moisture	5.4	5.3
Crude protein	27.3	42.7
Crude fiber	5.8	4.95
Total dietary fiber	59.1	38.9
Insoluble dietary fiber	53.9	33.45
Soluble dietary fiber	5.2	5.45
Crude protein: TDF ratio	0.46	1.09

YPSi, insoluble Yela ProSecure; YPS, Yela ProSecure.

#### 3.2.1 Gas production

**Figure 3:**
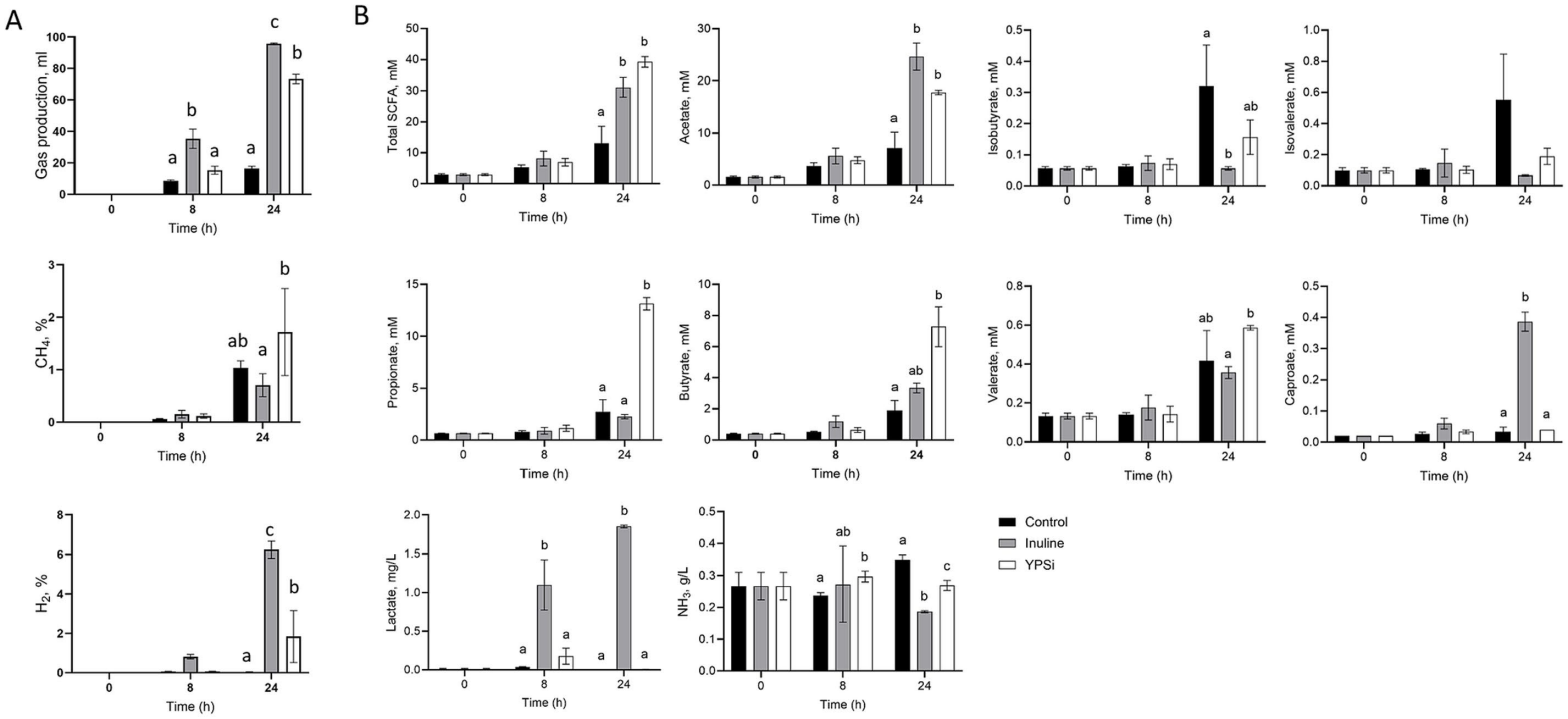
**(A)** *In vitro* effect of insoluble Yela ProSecure (YPSi) and of inulin on fecal microbial gases production. Adjusted means± SE. Different lowercase letters indicate significant differences between treatments for each time point through Tukey’s *post-hoc* test. **(B)** *In vitro* effect of insoluble Yela ProSecure (YPSi) and inulin on fecal microbial fermentative activity. Adjusted means ± SE. Different lowercase letters indicate significant differences between treatment for each time point through Tukey’s *post-hoc* test.

A significant interaction between time and treatment was obtained for total gas production (*p* < 0.001), showing an increase in production over time, with the highest production observed with inulin, followed by YPSi and the control condition (Figure 3A). A significant interaction between sampling time and treatment was obtained for CH_4_ and H_2_ production (*p* = 0.037 and *p* < 0.0001, respectively). CH_4_ percentage increased in all groups with time, but to a lesser extent in the inulin group at 24 h, while YPSi presented the highest CH_4_ value of 1.71% after 24 h. Conversely, the H_2_ percentage did not change in the control group over time, while it significantly increased in YPSi and, to a larger extent, in the inulin group at 24 h.

#### 3.2.2 SCFAs, BCFAs, and lactate

For all parameters, a significant interaction between time and treatment was observed (*p* < 0.01) (Figure 3B), and differences were discussed at *p* < 0.05. At 24 h, total SCFA and acetate production were not significantly different between YPSi and inulin treatments but were significantly higher than in the control. The significantly highest production of propionate, butyrate, and valerate at 24 h was observed in the YPSi group; regarding propionate, production was higher compared to the other two groups, while for butyrate and valerate, the inulin and the control groups displayed intermediate values, respectively. Significantly higher levels of isobutyrate were observed in control compared to inulin, while YPSi displayed an intermediate value at 24 h. Caproate concentration was strongly increased for inulin compared to control and YPSi, which remained stable over time. Lactate concentration was significantly higher in the inulin than in the other groups at both 8 and 24 h.

#### 3.2.3 Ammonia

A significant interaction between treatment and time was obtained (*p* = 0.005, Figure 3B). The concentration of ammonia decreased over time with inulin, while it increased with the control and remained stable with YPSi. At 24 h, a significantly lower concentration was achieved with inulin and was the highest in the control group.

#### 3.2.4 Bacteria count

Statistics were not performed on these data, as numerations were done in duplicate only. Overall, bacterial counts increased with fermentation time. Total anaerobes count was slightly higher in the inulin than in the YPSi group at 8 h, but at 24 h, it was higher in YPSi (Table 4). Total facultative anaerobes count was the highest with inulin at 8 h. At 24 h, counts were very similar for inulin and YPSi and higher than in the control group. Enterobacteria count increased in all groups at 8 h and was more elevated in the inulin group. At 24 h, it dropped dramatically in the inulin group, and YPSi exhibited a slightly higher count than the control. Finally, the count of lactic acid bacteria was the lowest in the control group at 8 h (8.91 ± 0.01 log_10_ CFU/g) and 24 h (8.9 ± 0.1 log_10_ CFU/g), while inulin and YPSi groups presented similar lactic acid bacteria counts at 24 h.

**Table 4:** Bacterial count in samples collected after 8 and 24 h of *in vitro* fermentation (log_10_ CFU/g of feces, means ± SD).

Item	0 h	8 h			24 h		
		Control	Inulin	YPSi	Control	Inulin	YPSi
Total anaerobes	8.18 ± 0.15	9.97 ± 0.16	10.29 ± 0.16	9.67 ± 0.13	10.11 ± 0.26	10.52 ± 0.00	11.43 ± 0.11
Total facultative anaerobes	8.36 ± 0.02	9.10 ± 0.04	10.25 ± 0.15	9.54 ± 0.02	9.29 ± 0.01	9.48 ± 0.06	9.50 ± 0.01
Enterobacteria	6.61 ± 0.07	8.92 ± 0.09	9.43 ± 0.01	9.21 ± 0.06	9.03 ± 0.02	4.89 ± 0.05	9.19 ± 0.03
Lactic acid bacteria	8.32 ± 0.01	8.91 ± 0.01	9.87 ± 0.19	9.45 ± 0.04	8.9 ± 0.07	9.56 ± 0.02	9.50 ± 0.00

### 3.3 Effects of YPS inclusion in the diet of piglets during post-weaning on performances

There was no effect of sex nor interactions on any of the measured parameters; therefore, those effects were removed from the model. A significant difference in BW was observed on day 18 (*p* < 0.001) and at the end of the experiment (*p* < 0.05), with the piglets in both YPS treatments being heavier than CON piglets (Table 5). A higher ADFI was observed between weaning and day 18 (*p* < 0.01) and overall (*p* < 0.05) in both YPS treatments, in addition to a trend between days 19 and 40 (*p* = 0.076). A numerically higher ADG was also noted between days 19 and 40 in those groups. Finally, there was a significant difference in FCR between weaning and day 18 (*p* < 0.001), with piglets in both YPS treatments displaying a better conversion compared to piglets in the CON treatment.

**Table 5:** Effect of including 2.5 and 6% of Yela ProSecure on the zootechnical performance of weanling piglets.

Variable		CON	YPS 2.5	YPS 6	SEM	*p*-value
BW (kg)	IBW	7.30	7.30	7.29	0.150	-
	Day 18	11.64^b^	13.61^a^	13.15^a^	0.160	< 0.001
	Day 40	23.98^b^	27.02^a^	26.11^a^	0.417	0.025
ADFI (kg/d)	Days 0–18	0.440^b^	0.547^a^	0.506^a^	0.010	0.002
	Days 19–40	0.987^y^	1.109^x^	1.094^x^	0.022	0.076
	Overall	0.752^b^	0.868^a^	0.842^a^	0.016	0.021
ADG (kg/d)	Days 0–18	0.241^b^	0.351^a^	0.325^a^	0.009	< 0.001
	Days 19–40	0.514	0.559	0.543	0.014	0.431
	Overall	0.397^b^	0.470^a^	0.448^a^	0.010	0.025
FCR	Days 0–18	1.864^b^	1.568^a^	1.562^a^	0.027	< 0.001
	Days 19–40	1.943	1.991	2.014	0.033	0.684
	Overall	1.908	1.852	1.881	0.021	0.547

^a,b^Different superscripts indicate significant differences between means. ^x,y^: Different superscripts indicate a trend to a significant difference between means. CON, control; YPS, Yela ProSecure; SEM, standard error of the mean; BW, body weight; ADFI, average daily feed intake; ADG, average daily gain; FCR, feed conversion ratio; IBW, initial body weight. Treatments, CON, control; YPS 2.5, standard diet + 2.5% YPS between days 3–18; YPS 6, standard diet + 6% YPS between days 3–18.

Among the feces collected for microbiota analysis, the odds of having “diarrheic score” decreased compared to the control when YPS was added, especially at the 2.5% inclusion rate (odds ratio = 0.227; *p* = 0.006; Supplementary Table 4). A time effect was also observed with smaller odds on day 40 when compared to day 4 (Odds ratio = 0.310; *p* = 0.033).

### 3.4 Effects of YPS inclusion in the diet of piglets during post-weaning on their fecal microbiota

The YPS effect on the fecal microbiota was investigated after 2 days and 16 days of treatment (i.e., on days 4 and 18 corresponding to the beginning and the end of phase 2). The potential lasting effect of YPS was assessed at the end of phase 3 (i.e., on day 40 after 19 days of withdrawal from the diet). After processing, there were 13,009 ± 1,458 (mean ± SD) sequences per sample and a total of 3,446 ASVs generated (417 bp mean length, Supplementary Table 6).

#### 3.4.1 Effect of YPS on microbial diversity

The alpha diversity was neither impacted by time nor by diet (Supplementary Figure 1). However, time significantly impacted the microbiota composition as evidenced by the analyses of the beta diversity (Supplementary Figure 2; date effect in adonis test *p* < 0.001; R2 = 0.09 R^2^ = 0.06, and R^2^ = 0.08 for Bray–Curtis dissimilarity, unweighted UniFrac, and weighted UniFrac, respectively). The microbiota beta diversity gradually decreased over time from day 4 to day 40 (Supplementary Figure 2). The effects of YPS on the overall microbiota composition are illustrated in Supplementary Figure 3. According to the analyses of the Bray–Curtis dissimilarity, both doses of YPS significantly impacted the microbiota composition on day 4 post-weaning when compared to the composition obtained in the control group (*p* < 0.05, R^2^ = 0.06 and R^2^ = 0.05 with 2.5 and 6% YPS, respectively). On day 18, only the lower dose had a significant effect (*p* < 0.001, R^2^ = 0.09), whereas a trend was observed with the higher dose (*p* = 0.06, R^2^ = 0.05). The effect of YPS was not detectable anymore on day 40. However, using UniFrac distances to evaluate the beta diversity, the effect of YPS was found to be significant only on day 18, with unweighted UniFrac distance.

#### 3.4.2 Effects of YPS on the fecal microbial composition

**Figure 4:**
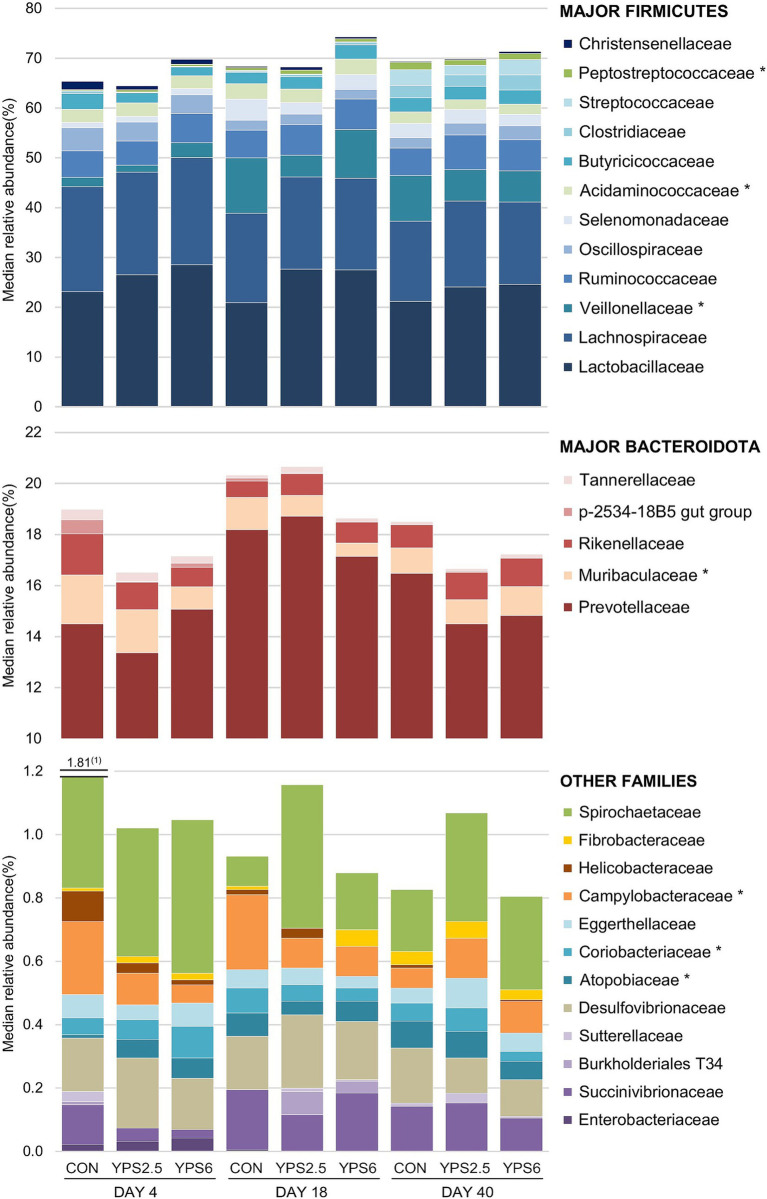
Bacterial composition at the family level of fecal samples collected at 4, 18, and 40 days post-weaning from piglets receiving control diet (CON), YPS at 2.5% (YPS2.5), or YPS at 6% (YPS6), with n = 17 per group. Only families with a median relative abundance >0.1% (Firmicutes), >0.3% (Bacteroidota), or >0.03% (other phyla) in at least one group are shown. * indicates *p* < 0.05 for a treatment effect for at least one date of sampling. ^(1)^ *Spirochaetaceae* median relative abundance.

Fecal microbiota composition is presented at the phylum and family levels (Supplementary Figure 4; Figure 4). As described previously in weanling piglets from various breeds, the most abundant phyla detected in fecal samples, no matter the date of sampling, were *Firmicutes* (76% ± 7%, mean ± SD), mostly represented by *Lactobacillaceae* (24% ± 9%) and *Lachnospiraceae* (19% ± 4%) families, followed by *Bacteroidota* (20% ± 6%), especially *Prevotellaceae* family (16% ± 6%). The effect of YPS groups on the relative abundance of microbial genera and families was subtle (Supplementary Tables 7, 8). The relative abundances of *Veillonellaceae* and *Muribaculaceae* were decreased in YPS groups on day 18 compared to control, whereas *Acidaminococcaceae*, *Peptostreptococcaceae*, *Erysipelotrichaceae*, *Atopobiaceae,* and *Coriobacteriaceae* exhibited higher relative abundances on days 4 or 18, regardless of the dose administrated (Figure 4; Supplementary Table 7). The lower level of *Campylobacterota* (*p adj.* < 0.05), represented by *Campylobacteraceae* and *Helicobacteraceae* families, was evidenced in YPS groups on days 4 and 18 compared to the control group (Supplementary Figure 4; Supplementary Table 9). The effect was dose-dependent, with a lower level in the group receiving the higher dose of YPS. There was no further detectable effect of the YPS treatment on day 40.

**Figure 5:**
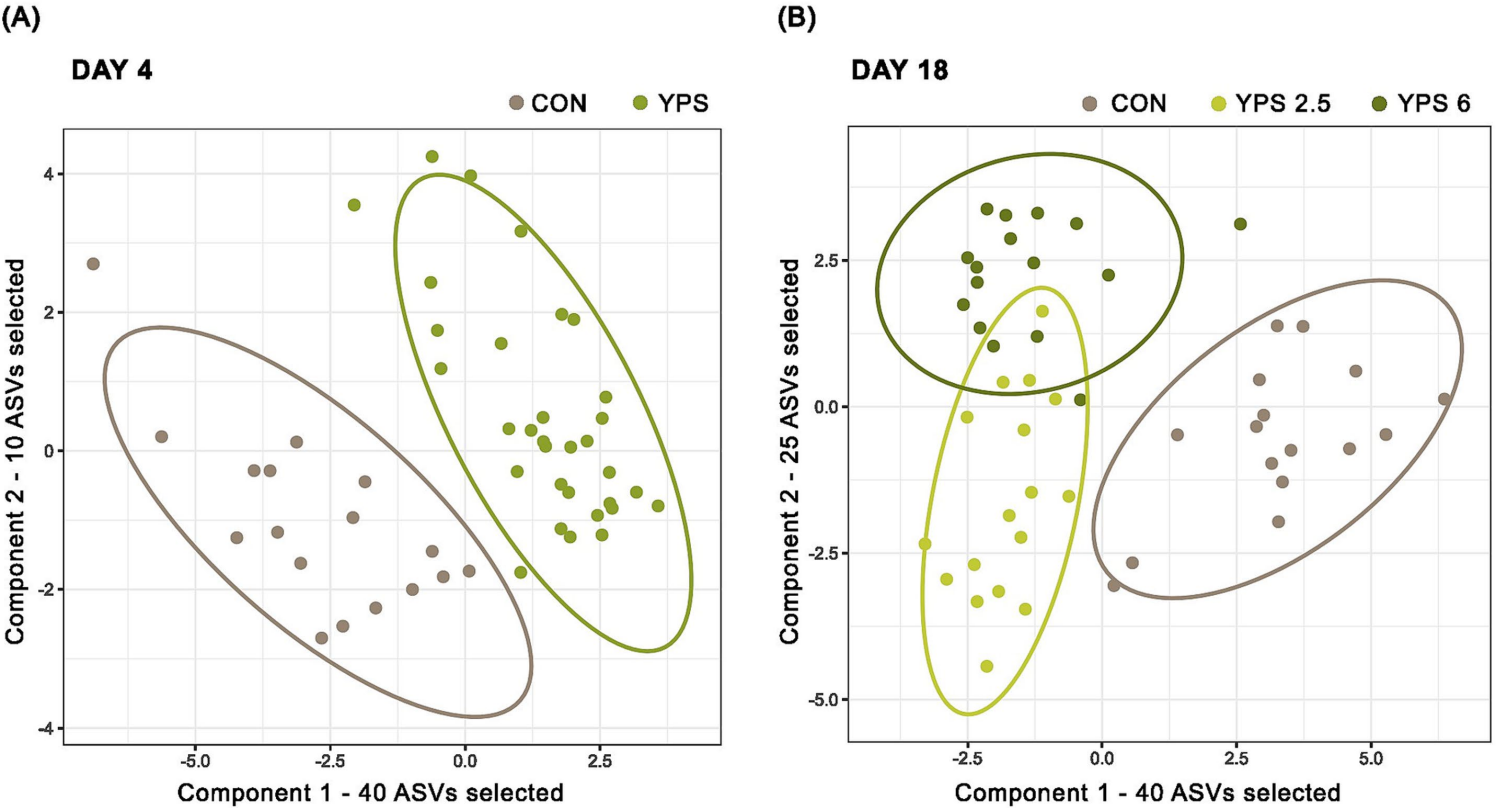
Visualization of the results of sPLS-DA applied on samples from day 4 and day 18. **(A)** sPLS-DA model with two components; 40 and 10 ASVs, respectively, were selected for the components to discriminate two groups of samples: piglets receiving a control diet (CON) and piglets receiving YPS at 2.5% or 6% (YPS). **(B)** sPLS-DA model with two components; 40 and 25 ASVs, respectively, selected for the components to discriminate three groups of samples: piglets receiving control diet (CON), YPS at 2.5% (YPS 2.5), and YPS at 6% (YPS 6); 0.8 confidence ellipses are represented.

**Figure 6:**
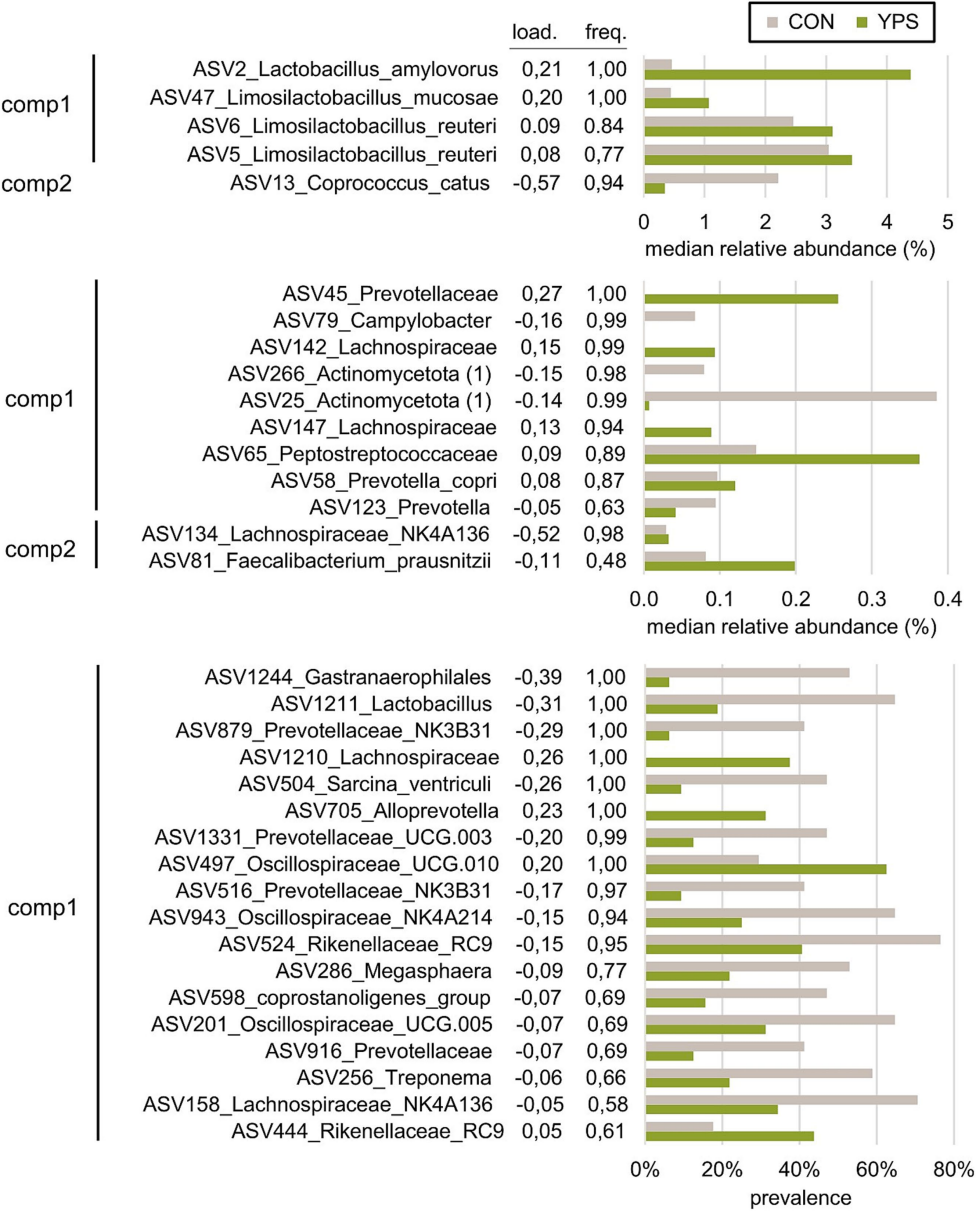
Most discriminative ASVs selected with the sPLS-DA model applied on day 4 samples to discriminate the CON group (n = 16) and YPS group (n = 32). **(A)** ASVs with median relative abundances >0.4% in at least one group. **(B)** ASVs with median relative abundance between 0.05 and 0.4% in at least one group. **(C)** ASVs with median relative abundance <0.05% in both groups. The selected ASVs are grouped according to the component of the sPLS-DA (comp1 and comp2) and ranked according to their loading value—i.e., their importance in the sPLS-DA model. Load. = loading value; freq.: frequency of ASV selection calculated with the iterative cross-validation of the model. Taxonomic annotations are given at the family, the genus, or the species level when relevant.

**Figure 7:**
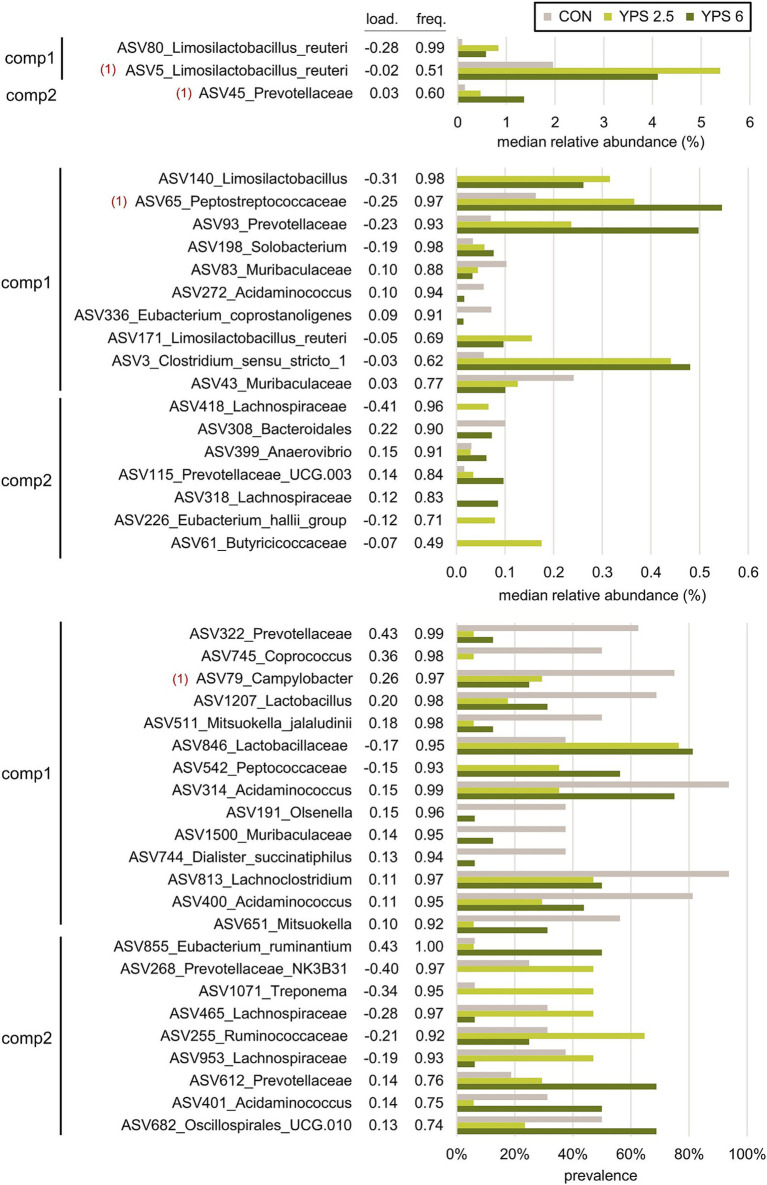
Most discriminative ASVs selected with the sPLS-DA model applied on day 18 samples to discriminate the CON group, YPS 2.5 group, and YPS 6 group (n = 16 per group). **(A)** ASVs with median relative abundances >0.6% in at least one group. **(B)** ASVs with median relative abundance between 0.05 and 0.6% in at least one group. **(C)** ASVs with median relative abundance <0.05% in all groups. The selected ASVs are grouped according to the component of the sPLS-DA (comp1 and comp2) and ranked according to their loading value—i.e., their importance in the sPLS-DA model. Load. = loading value; freq.: frequency of ASV selection calculated with the iterative cross-validation of the model. Taxonomic annotations are given at the family, the genus, or the species level when relevant (1). ASVs were also found discriminative between CON and YPS groups on day 4.

A PLS-DA was carried out to further investigate the effect of YPS treatment on the ASV composition on day 4 and day 18. On day 4, the PLS-DA model did not allow discrimination between the two doses of YPS (calculated error rate > 50%). The two groups of YPS samples were thus combined, which improved the performance of the discrimination with the control group (28.3% error rate). On day 18, a validated model was allowed to discriminate between the control and the two doses of YPS (33.4% error rate). The discriminant analyses confirmed the effect of YPS on the microbiota composition on day 4 and day 18, and the most discriminative ASVs were selected using sPLS-DA for both time points (Figure 5). The performance of the model was optimized when 50 and 65 ASVs were selected on day 4 and day 18, respectively (Table 6). A full list of the selected ASVs is provided in Supplementary Tables 10 and 11. Among the most discriminative ASVs identified on day 4 (Figure 6), two were annotated as *Lactobacillus amylovorus* and *Limosilactobacillus mucosae* (ASV2 and ASV47). These ASVs are among the 10 most abundant ASVs on day 4 in YPS groups. Three other discriminative ASVs belonged to *Lactobacillaceae* family: two abundant ASVs annotated as *L. reuteri* (ASV5 and ASV6) and associated with the YPS group, and a rare *Lactobacillus* (ASV1211, detected in less than half of the samples, 0.20% maximal relative abundance) highly discriminative of the control group. The most discriminative ASV on component 2 was annotated as *Coprococcus catus*, with a relative abundance of more than 2% in control vs. less than 0.5% in the YPS groups. Other discriminative ASVs associated with YPS treatment were annotated as unknown *Peptostreptococcaceae* (ASV65), unknown *Lachnospiraceae* (3 ASVs), and *Faecalibacterium prausnitzii* (ASV81). Discriminative ASVs associated with the control group include ASVs annotated as *Campylobacter* (ASV79), unknown *Gastranaerophilales* (ASV1244), *Sarcina ventriculi* (ASV504), *Megasphaera* (ASV286), and *Treponema* (ASV256). Discriminative ASVs belonging to *Prevotellaceae* family (10 ASV) or the genus *Rickenellaceae RC9 gut group* (3 ASVs) were enriched in one or the other YPS groups (Figure 6; Supplementary Table 10). Only 4 of the ASVs discriminative on day 4 were also selected by the sPLS-DA model applied on day 18 (ASV5, ASV45, ASV65, and ASV79, Figure 7). Nevertheless, several discriminative ASVs selected on day 18 exhibited similar taxonomic annotations to the ones selected on day 4. In particular, three ASVs assigned to *Limosilactobacilli* were drastically enriched in the two YPS groups when compared to the control group. The taxonomic annotation *Muribaculaceae* (2 ASVs), *Acidaminococcus* (4 ASVs), *Mitsuekella* (2 ASVs), *Olsenella,* or *Dialister*, not identified on day 4, were found associated with the control group on day 18 (Figure 7; Supplementary Table 11). ASVs selected on the second component of the sPLS-DA model and associated with the higher dosage of YPS are annotated as unknown *Bacteroidales* (ASV308), *Anaerovibrio* (ASV399), *Eubacterium ruminantium* (ASV855), *Oscillospirales UCG-010 group* (ASV682), and *Acidaminococcus* (ASV682). On the other hand, *Eubacterium halli group* (ASV226) or *Butyricoccaceae* (ASV61) annotations were highlighted in the group of samples from piglets receiving YPS at 2.5%.

**Table 6:** Evaluation of the performance of the models to discriminate the fecal microbiota of piglets receiving YPS or not on days 4 and 18.

Day	Treatment comparison	ASVs number in dataset	Model performance		ASV selected		
			PLS-DA	sPLS-DA	Comp1	Comp2	Total
Day 4	CON vs. YPS 2.5 vs. YPS 6	543	50.4% (±20.1%)	-	-	-	-
	CON vs. YPS	543	28.3%	35.9%	40	10	50
Day 18	CON vs. YPS 2.5 vs. YPS 6	527	33.4% (±15.5%)	35.3% (±12.3%)	40	25	65

The mean error rates of the treatment group included in the model are given (± SD, when three treatment groups are included in the model).

#### 3.4.3 PLS regression between microbiota data and performance

PLS regressions were carried out to highlight ASVs of potential interest that correlate with the ADG of each feeding phase. PLS models were validated between the day 4 microbial dataset and phases 1 and 2 ADG, as well as between the day 18 microbial dataset and phase 3 ADG, despite a low predictive power (Table 7). On the contrary, the performance of the day 4 microbial dataset to predict phase 3 ADG was too weak to be further investigated (Q2 < 0). Sparse PLS regression allowed for the selection of 150 ASVs on day 4 and 70 ASVs on day 18 while maintaining the performances (Supplementary Tables 12, 13). We highlighted a lower correlation on day 4 than on day 18 for ADG phases 1 and 2, and ADG phase 3, respectively (0.69 vs. 0.79, Supplementary Figure 5).

**Table 7:** Evaluation of the performance of the PLS used to evaluate the ASVs that may predict the Average Daily Gain (ADG) of the piglets.

Day	Variable compared	ASVs number in dataset	Model performance (one component)		ASV selected	Correlation
			PLS	sPLS		
ASVs day 4	ADG days 0–18	639	R2 = 0.1 ± 0.01 Q2 = 0.04 ± 0.03	R2 = 0.08 ± 0.02 Q2 = −0.02 ± 0.04	150	0.69
	ADG days 19–40	639	R2 = 0.04 ± 0.01 Q2 = −0.11 ± 0.03	-	-	-
ASVs day18	ADG days 19–40	574	R2 = 0.25 ± 0.02 Q2 = 0.24 ± 0.02	R2 = 0.24 ± 0.03 Q2 = 0.23 ± 0.04	70	0.79

#### 3.4.4 Functional inference

**Figure 8:**
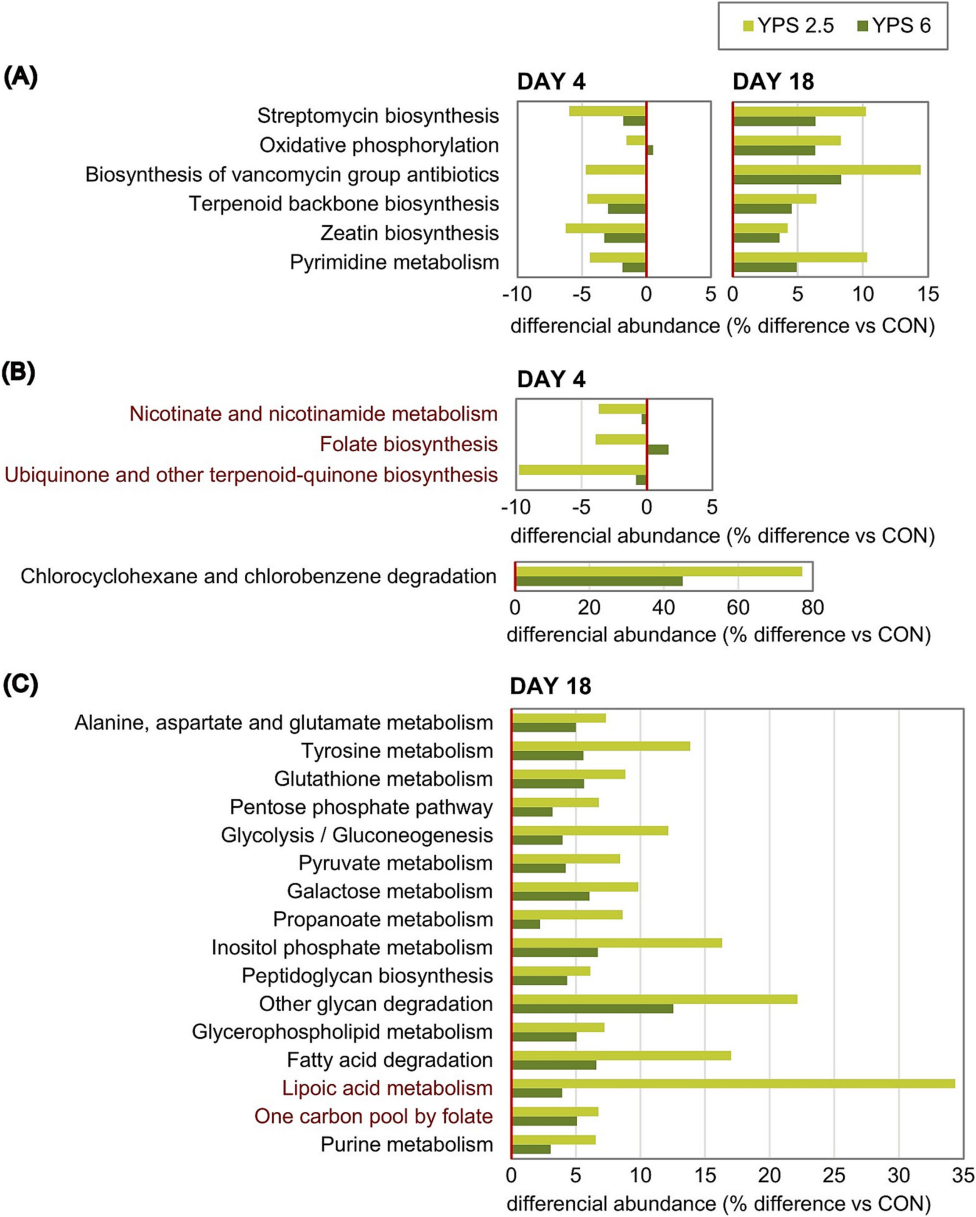
Predicted metabolism pathways are differentially abundant. Adjusted *p* < 0.1 for date x treatment effect (ANOVA test) and *p* < 0.05 for *post-hoc* treatment effect. **(A)** Pathways with significantly different abundances on day 4 and day 18. **(B)** Pathways with significantly different abundances only on day 4. **(C)** Pathways with significantly different abundances only on day 18. Pathways related to the metabolism of cofactors and vitamins are highlighted in red.

The abundance of the KEGG molecular function pathways was predicted based on PICRUSt2 analysis and the KO database. The 134 inferred pathways were classified into subclasses related to metabolism, cellular processes, environmental information processing, genetic information processing, human diseases, and organismal systems (Supplementary Table 14). A total of 90 pathways were related to metabolism, mainly including subclasses of pathways related to the metabolism of carbohydrates, cofactors and vitamins, amino acids, terpenoids and polyketides, and lipids (Supplementary Figure 6). YPS treatment significantly affected the inferred abundance of 40 pathways (adjusted *p* < 0.1), including 26 pathways related to metabolism and 11 related to genetic information processing (Supplementary Table 14). The maximal difference between the control group and the YPS group was surprisingly evidenced for the chlorocyclohexane and chlorobenzene degradation subclass, exhibiting on day 4 a predicted abundance 80% higher in the YPS 2.5 group than in the control group (Figure 8). This result should be, however, interpreted with caution given the very low abundance of this pathway. Despite being significant (adjusted *p* < 0.1), the effect of YPS treatment on pathway abundance was overall minor, with more pathways impacted on day 18, particularly with the lower dose of YPS. Interestingly, five pathways related to the metabolism of cofactors and vitamins, out of 12 identified in this subclass, were found to be differentially abundant (Figure 8, in red). More specifically, pathways related to vitamin B3 (nicotinate and nicotinamide), B9 (folate), and coenzyme Q10 (ubiquinone) metabolism or biosynthesis were less abundant in the YPS group on day 4. On the other hand, a noticeable increase in the abundance of lipoic acid metabolism and the one-carbon pool by folate pathways was observed in the YPS 2.5 group on day 18. Other differential pathways were related to the metabolism of amino acids (alanine, aspartate, glutamate, and tyrosine) glutathione, carbohydrates, including propanoate, lipids, including glycerophospholipid, as well as terpenoids and polyketides. All pathways identified as differential on day 18 exhibited a higher predicted abundance in the YPS group.

## 4 Discussion

There is an increasing demand for alternative protein sources in animal nutrition. Important selection criteria for novel protein sources in piglet diets consist of the nutritional value of the protein (AA digestibility notably) to improve the efficiency of protein utilization, the palatability to support optimal feed intake, the functional role to sustain animal wellbeing and digestive health, and the sustainability of the production of the protein source.

Among the proposed new candidates (insect, algae, microbial biomass, plant, and genetically modified plants), yeast proteins are promising candidates because of their scalability, sustainability, the absence of competition between food and feed, and their nutritional and functional properties. A hydrolyzed yeast was characterized *in vitro* by comparing its protein digestion kinetics with other traditional protein sources used in piglets’ diets and by assessing its AA digestion kinetics for 48 h. Then, the impacts of the insoluble fraction on the fecal microbiota activity and composition were studied, and the benefits observed were further appraised *in vivo* on piglets’ performance and gut microbiota.

*In vitro characterization of YPS in terms of kinetic of amino acids absorption and fermentative activity…*

Currently, feed formulation approaches typically do not account for protein digestion kinetics when assessing the nutritional value of a protein source (12), although it gives valuable information about the time when the AA and low molecular weight peptides from the proteins are available for absorption by the animals. Identifying the moment and site of absorption can help to synchronize energy and protein supply and likely benefit feed efficiency (12). Interestingly, YPS harbored a faster *in vitro* protein digestibility than SBM and FM, two other common protein sources used in swine, which are considered highly digestible. YPS and the specific FM used in our comparison displayed a higher *in vitro* protein digestibility than SBM after 48 h of digestion (93.8, 96.1, and 90%, respectively). In the first 3 h of digestion, YPS displayed the highest absorption compared to FM and SBM (87.3, 80.9, and 69.5%, respectively). The *in vitro* digestibility values obtained for SBM in this study were in line with previous studies (11, 12). Information about *in vitro* digestibility of FM in the literature is scarce and variable, mostly due to the different qualities of FM used (origin, production process, and crude protein content).

Values of AA digestibility in brewer’s yeast have been shown to be significantly affected by the processing methods of yeast products (31). Globally, YPS harbored highly digestible AA, with an *in vitro* digestibility higher than 75% except for His, Ser, Thr, and Tyr. Of note, Met and Cys, the two sulfur-AA, are fully absorbed after 3 h of *in vitro* digestion. The nutritional value of protein in feedstuffs not only depends on its AA content but also on its molecular structure characteristics (32). Recent studies have highlighted the importance of determining the relationship between molecular spectral band characteristics of proteins and their intestinal absorption (33). Bai et al. (34) showed that the molecular structure characteristics of feed proteins are closely related to their *in vitro* digestibility and solubility. It would be of interest to further analyze the molecular structure characteristics of the YPS proteins to bring more information on their nutritional value.

In an attempt to mimic the impact of the undigestible fraction of YPS reaching the colon on piglets´ fecal fermentative activity, an insoluble fraction of YPS (YPSi) was used in an *in vitro* model. In that study, YPSi supplementation to *in vitro* fecal medium promoted gas and SCFA production and influenced the total number of strict and facultative anaerobes. Inulin is a soluble fiber made of fructans, which has been described to stimulate microbial activity (35). When compared to inulin, the fermentative profile obtained with YPSi was modified with less lactate and acetate concentrations and more propionate, butyrate, and valerate concentrations. This might be related to cross-feeding between bacteria, notably the use of lactate to produce different SCFAs and acetate to produce butyrate. The results obtained with inulin as a positive control agreed with the literature (18). More specifically, it stimulates the growth of homo- and hetero-fermentative lactic acid bacteria, resulting in a strong production of lactate, acetate, and caproate. The latter has been shown to be produced from lactate oxidation to provide acetyl-CoA, which enters the elongation process to form butyryl-CoA and caproyl-CoA (36). Meanwhile, it induced a decrease in *Proteobacteria* growth, including *E. coli*, as well as in proteolytic activity and ammonia production.

The addition of YPSi in *in vitro* fermentations resulted in the growth of various functional groups of bacteria (total and facultative anaerobes, lactic acid bacteria, etc.), highlighting its potential to be used as a non-selective substrate for the microbiota. The fiber part of YPSi likely played a role in the production of SCFAs in our *in vitro* study. Based on the knowledge about the composition of the yeast cell wall, the composition of YPSi mainly consists of fermentable compounds, notably different types of fiber (soluble, insoluble chitin-glucan, mannoproteins fiber, etc.) and proteins or peptides cross-linked with glycans (37). The higher production of total SCFAs with YPSi may be at least partially explained by the insoluble fraction of proteins left available for the microbiota in this *in vitro* model. The AA profile of YPS revealed high contents of aspartate and glutamate, two acidic AAs that lead to the production of acetate and butyrate and the production of acetate and propionate, respectively (38). Similarly, lysine catabolism can result in butyrate and acetate production. The microbial metabolism of serine and threonine can result in the formation of a variety of products, including propionate, the ultimate product depending on the catabolic pathways used by bacteria in the ecosystem. According to the analysis of AA *in vitro* digestion kinetics of YPS, a high portion of the insoluble protein fraction of YPS is likely to be digested and absorbed in the upper gut. Nevertheless, these *in vitro* results suggest that a small portion of AA, which would be reaching the lower gut *in vivo* has the potential to modulate the microbiota composition and stimulate the production of SCFA.

Interestingly, we report a decrease in ammonia production associated with an increase in butyrate concentration after 24 h. *In vivo*, ammonium chloride at 20 mmol/L concentration has been shown to decrease the gene expression of monocarboxylate transporter 1 (*MCT1*) involved in butyrate uptake by colonocytes in the pig colon (39). Therefore, more efficient production and use of butyrate, together with weaker ammonia production, could occur when piglets are fed YPS, contributing to better performance, as seen in our *in vivo* study. It can be hypothesized that the supply of fiber provided by YPSi allows for optimizing the protein-to-fiber ratio for the production of SCFAs, and/or selective fermentation activity. Uerlings et al. (18) observed an increased SCFA and butyrate production during an *in vitro* fermentation of pig feces supplemented with inulin. This inulin-fermented media was then shown to increase the expressions of tight and adherent junction genes in IPEC-J2 cells (18). The highest production of SCFA and butyrate observed with YPSi *in vitro* could thus be an explanation for the decreased diarrheic score observed in the *in vivo* study.

In agreement with our results, two *in vitro* studies using human fecal *inocula* and chitin-glucan fiber in either a dynamic model (SHIME model) (40) or batch model (41) highlighted a propionogenic and butyrogenic effect and no big modification of lactate and ammonia production. Catalayud et al. (41) observed an increase in the relative abundance of butyrate- and propionate-producing bacteria, such as *Faecalibacterium prausnitzii* and *Roseburia intestinalis*. In addition, they showed effects on inflammation mitigation, epithelial barrier recovery, and anti-inflammatory cytokine production. Indeed, a consistent increase in the immunomodulatory cytokines IL-10 and IL-6 was depicted using co-culture of intestinal epithelium cells and macrophages subjected to an LPS challenge, incubated or not with supernatants from a batch digestive *in vitro* model. The production of butyrate, propionate, and other metabolites produced by bacteria are proposed to explain the positive and promising *in vitro* immunomodulation effects of such types of fiber.

*…agrees with in vivo effects on zootechnical performance and fecal microbiota of weanling piglets fed with YPS.*

In our study, we used Iberian × Duroc piglets. This genetic is less selected and fattier than the traditionally commercial white pigs, and its metabolism implies a lower efficiency in the conversion of feed to meat and, therefore, a decreased performance compared to white pigs. In this context, a complete N balance performed at the Consejo Superior de Investigaciones Científicas (CSIC, Granada, Spain) in Iberian × Duroc piglets between 10 and 27 kg of BW, fed diets with 16.0% crude protein, depicted an FCR of 2.06, with 489 g/d of growth and 1.004 kg/d of feed intake (personal information), which is in line with the values obtained in our study for these variables.

*In vivo,* the inclusion of YPS at either 2.5% or 6% improved BW and ADG throughout the study, even if the effect was more marked from weaning to day 18. This is in line with other works reporting an improvement in post-weaning performance when other hydrolyzed yeasts were used in the diets of piglets (8), with inclusion rates of 0.5 and 10% at the expense of SBM. These authors reported a positive dose–response relationship on zootechnical performance, crude protein digestibility, and nitrogen retention of piglets. In our study, we did not obtain such a dose–response relationship, as no difference was noticed in performance between the 2.5 and 6% inclusion rates. The discrepancy between the two studies could be related to the age of the piglets at weaning, older (18 days vs. 28 days in our study) and thus with a more mature digestive tract than in Boontiam et al. (8), to the hydrolysis process of the yeast used, and to the dosage. Based on the current results, inclusion rates of YPS between 2.5 and 6% in an SBM-based diet seem compatible with the optimal growth performance of weanling piglets.

The voluntary feed intake of piglets was also improved with the inclusion of YPS, partially contributing to the better growth performance observed. Voluntary feed intake after weaning is a crucial parameter for growth, while being highly variable among individuals, especially during the first week post-weaning. Voluntary feed intake depends on a great deal of factors (42), among which is the palatability of the diet, which could have been improved in our study with the addition of YPS. Indeed, protein and AA contents are recognized as being important in the chemosensory system of piglets (43). More precisely, the umami taste has been related to enhanced feed intake in young piglets (44). This taste involved seven AAs including Ala, Asn, Asp., Glu, Gln, Pro, and Thr (45). Those AAs are well-represented in YPS, and due to the hydrolysis of the product, they are quickly available in the gastrointestinal tract (as demonstrated in the *in vitro* digestion kinetic analysis) so that they could contribute to enhancing the palatability of the diet. This result suggests the importance not only to offer a balanced diet based on the most limiting AA but also to consider homeostatic and hedonic aspects when adjusting AA in the diet to optimize growth and feed intake. Of note, the AA sensors probably regulate the secretion of different gut peptides, which can modulate satiety signals (46, 47). Thus, it could be interesting for future research to look at the effect of yeast protein sources on the secretion of glucagon-like peptide 1, peptide YY, cholecystokinin, ghrelin, and other gut peptides.

The addition of YPS resulted in a better FCR from weaning to 18 days post-weaning, suggesting that the higher voluntary feed intake is not the only explanation for the better growth. The high N digestibility and the fast availability of each AA observed in our *in vitro* characterization could contribute to explaining the enhanced performance. Indeed, there is *in vivo* evidence of an increased digestibility of crude protein with yeast supplementation (4, 48). The high AA availability in the earlier phase of the gastrointestinal tract (up to 3 h), as observed *in vitro*, presumably ensured an early and synchronic uptake of dietary AA. Interestingly, recent findings showed that the kinetics of AA released from dietary protein have substantial effects on protein synthesis in muscle (49, 50), suggesting that AA release kinetics are closely related to N deposition in animals. In particular, branched-chain AA and glutamine are able to activate the mTOR pathway involved in the long-term metabolic control of feed intake and stimulation of protein synthesis (51). Based on the *in vitro* results associated with better performance *in vivo*, we can hypothesize that adding YPS to the diet could favor synchronicity of AA absorption, leading to better muscle protein synthesis. This will deserve more attention and further research to measure N retention *in vivo*, which is a parameter of uttermost importance for the sustainability of animal production.

Improved performance could also be related to the effect of YPS on the gut microbiota of piglets. The observed trend to decrease the odds of having a diarrheic score may suggest improved gut health. Among the noticeable microbial modifications, the increase in relative abundance of different *Lactobacillaceae* is of interest in this context. *L. amylovorus* is an abundant species in the small intestine of pigs (up to 20% relative abundance) and has been reported to exert carbohydrate metabolic functions and beneficial effects by modulating gut immunity and inflammation (52, 53). Importantly, the growth of this species is stimulated in a peptide-rich substrate as demonstrated in the elegant *in vitro* study of Jing et al. (52). The authors found that the peptide-rich substrate promoted a dominance of lactobacilli, and more precisely of *L. amylovorus*, associated with an increase in lactate and butyrate production due to a concomitant rise in the relative abundance of *Megasphaera*. Peptide-rich substrates obviously had more impact in jejunum and ileum, which are major sites of absorption, than in the colon (54). Whether the increase in *L. amylovorus* in feces in the current study reflects the one in the small intestine remains to be further elucidated with the collection of *in situ* samples. However, the presence of *L. amylovorus* in the feces of piglets from the YPS group could be driven by AA utilization in the upper tract, highlighting the important role of peptide-rich diet—often underestimated—in the enrichment of lactic acid bacteria in the gastrointestinal tract of piglets. In addition, the production of bacteriocins by *L. amylovorus* has been documented (55), and these bacteriocins inhibit *Helicobacter pylori* growth (56). Consistently with this, we observed an increase in a putative *L. amylovorus* (ASV2) concomitantly to a decrease in *Campylobacteraceae* relative abundance in the YPS groups on day 4. Another ASV annotated as *Limosilactobacillus mucosae* was also found discriminative in favor of the YPS group. Interestingly, the microbiota of piglets with a diarrheic fecal score was found to be devoid of *L. mucosae* compared to healthy piglets, and *L. mucosae*-derived extracellular vesicles were able to alleviate diarrheal disease symptoms caused by ETEC K88 by regulating macrophage phenotypes (57). On top of that, *L. mucosae* is well-described to produce gamma-aminobutyric acid (GABA), a neurotransmitter that can increase feed intake (51). Future studies targeting the functional roles of those specific lactobacilli in reshaping the gut microbiota, complexifying the microbial interconnections, and existing crosstalk with the host could be of great interest.

Although in lower relative abundances, other ASVs found to be discriminant or different between the control and YPS groups remain of interest and corroborate the first observations performed during our *in vitro* experiments. First, butyrate-producing bacteria, such as *F. prausnitzii*, as well as *Lachnospiraceae*-related ASVs or *Eubacterium ruminantium,* were found to be enriched in the YPS group, while *Coprococcus catus*, a potential butyrate producer discriminated the control group. The latter has been found to be more abundant in the colon of pigs fed a low-fiber diet (58), while the others are strictly anaerobic, fermentative, pH, and O_2_ sensitive, and can degrade non-starch polysaccharides to produce acetate and butyrate. This agrees with the fact that YPS supplies a substantial amount of fiber that can be used by the microbiota. Of note, *F. prausnitzii* has been associated with better mitigation of inflammation and a more robust weaning process. Relative abundances of *Acidiminococcaceae* and *Peptostreptococcaceae* were also found enriched in the YPS groups. Those bacteria produce BCFAs and possess the hydroxyglutamate pathway, leading to the production of acetate, butyrate, and CO_2_ from AA. Finally, several *Prevotellaceae*-related ASVs, among which *P. copri*, also differentiated the two diets. These bacteria can metabolize carbohydrates and proteins and thus have wide metabolic capacities but are generally recognized as propionate producers, which can corroborate with the increase in propanoate metabolism pathway abundance observed when performing functional inference. In humans, *Prevotella*-dominated microbiomes have been shown to occur in populations consuming agrarian fiber-rich diets. Interestingly, among the top 40 ASVs predicting the ADG on day 4, four (one *Prevotellaceae*-, one UGC-10-, one *E. ruminantium*-, and one *Lachnospiraceae*-related ASV) found as positively discriminant for the YPS group were positively associated with ADG; while four (one *Campylobacter*-, one *Megasphaera*-, one NK4A214-, and one *Lachnospiraceae*-related ASV) associated with the control group were negatively linked to the ADG. These different results may suggest a modulation of the intestinal micro-environment, leading to a shift in the microbial ecosystem and to more complex relationships among bacteria. In turn, that could improve microbiota resilience and contribute to better performance just after weaning.

Microbial composition is of importance, but it is known that there are functional overlaps between bacteria to produce a set of metabolites. Those metabolites can be used either by other bacteria through cross-feeding or by the host, so that they may impact feeding behavior, gut health, and immunity of the host, reshaping the gut microbiota and contributing to better performance and health. We thus performed a preliminary functional inference analysis to see if some microbial functional capabilities were modulated when adding YPS to the diet of weanling piglets. Some impacted subclasses were related to the metabolism of terpenoids and polyketides, as well as the cofactors and vitamins. Unlike dietary vitamins, which are mainly absorbed in the proximal part of the small intestine, the uptake of microbial vitamins predominantly occurs in the colon, so the microbiota-produced vitamins may contribute to the systemic vitamin levels and especially to the homeostasis of the vitamins in the localized epithelial cells (59). Unabsorbed vitamins can also be used by the microbiota. In the context of the high availability of B vitamins in the upper gut, we can hypothesize that leftovers may reach the lower gut, favoring the growth of bacteria that do not produce these vitamins. This could indeed explain the lower level of vitamin biosynthesis pathways observed on day 4. Niacin and lipoic acid exhibit potent antioxidant and anti-inflammatory properties, acting as a modulator of intestinal barrier function and bacterial endotoxin production. A deficiency may result in diarrhea (60). Folate is involved in many important pathways for the host, especially in DNA replication, repair, and methylation, and is required by fast-proliferating cells (59). Moreover, the one-carbon pool by the folate pathway allows the conversion of folate into its active form, tetrahydrofolate (THF). It is a crucial metabolic pathway that is essential for several biochemical processes, including DNA synthesis, essential amino acid metabolism, and methylation reactions. Ubiquinone and other terpenoid-quinone are usually referred to as hydrocarbon or terpenoid derivatives, and their oligomers, such as coenzyme Q10, squalene, farnesol, vitamin A, E, and K, are cofactors involved in many reactions and may be related to improvement in zootechnical performance (61). Glutathione metabolism was also modulated and is generally considered to be related to the promotion of cell redox balance (62). It is also interesting to note that the antimicrobial production pathways were affected by the diet, even if it is difficult to predict to which extent those can modulate the microbiota composition. The subclass of glycan degradation and biosynthesis was also differentially affected by the diet. As stated above, YPS contains fiber, which can induce upregulation of glycan pathways. Several pathways related to AA, carbohydrate, and lipid metabolism were also upregulated in the YPS group, especially on day 18, corroborating the hypothesis of a different community structure and more complex relationships among bacteria with YPS supplementation. However, PICRUSt2 analysis is only a predictor of metagenomic function, and metabolomic approaches would be preferred to identify factual changes in the metabolic function of the gut microbiota.

To conclude, hydrolyzed yeast appears as a promising ingredient with a nutritional and functional potential for use in piglet diets. Our complementary *in vitro* and *in vivo* approaches provided evidence that adding YPS to the diet of weanling piglets increased pig growth performance, probably due to nutrient absorption in the small intestine and its functional role on gut microbiota. Those results suggest complex interconnections between host and microbiota and emphasize the need to consider the holobiont theory when formulating a diet. Further research is required to continue to understand the importance of gastric emptying, small intestinal and colon microenvironments on performance, feeding behavior, gut barrier, and immune system of piglets fed yeast-based ingredients. In particular, *in situ* collection of intestinal samples and mucosa could be useful to validate the *in vitro* dynamic of protein and AAs, to objectivize an effect on the gut barrier and on interrelationships among bacteria. In addition, a better understanding of incretin patterns with the supply of palatable oligopeptides could highlight some potential gut–brain relationships to explain the effect on feed intake, which is a crucial outcome for weanling piglets. Finally, it will be helpful to calculate the nitrogen balance obtained when using such an ingredient, especially to assess its sustainable potential. Overall, new microbial-based raw materials represent a fantastic opportunity to meet the zootechnical, sustainable, and welfare requirements of modern breeding.

## Data Availability

The original contributions presented in the study are publicly available. This data can be found here: https://www.ncbi.nlm.nih.gov, accession number: PRJNA1284882.
